# CAR T cells for hematological malignancies

**DOI:** 10.1172/JCI177160

**Published:** 2024-01-16

**Authors:** Barbara Savoldo, Natalie Grover, Gianpietro Dotti

**Affiliations:** 1 Lineberger Comprehensive Cancer Center,; 2 Department of Pediatrics,; 3 Department of Medicine, and; 4 Department of Immunology, University of North Carolina at Chapel Hill, Chapel Hill, North Carolina, USA.

## An advanced treatment for hematological malignancies

For decades, the success of allogeneic hematopoietic stem cell transplant has highlighted the role of T cells in eliminating hematological tumors. This proof of concept pioneered the practice of the ex vivo generation and adoptive transfer of tumor-specific T cells (ATC), which became an effective modality, for example, for virus-associated diseases, like Epstein-Barr-virus-associated post-transplant lymphoproliferative disorders (1).

A remarkable advance to the ATC field was pioneered by Eshhar and colleagues in 1993, when, rather than replicating the T-cell receptor (TCR)-mediated recognition of tumor molecules, which requires antigen processing and presentation through major histocompatibility complex (MHC) molecules, they generated chimeric antigen receptors (CAR) that target unprocessed antigens expressed on the cell surface of tumor cells in an MHC unrestricted fashion (2). Providing T cells with the ability to engage targets via an antibody-derived single-chain-variable-fragment (scFv) linked to the intracellular T-cell signaling domains was game changing because it overcomes HLA barriers and opens a whole new antigen repertoire for tumor recognition. While the first clinical application of CAR-T cells was for treatment of patients with HIV (3), this approach gained the most traction in hematological malignancies and was driven by the availability of lineage restricted antigens homogeneously expressed by hematological tumors. Specifically, targeting B-cell malignancies offered a much-needed route to success (4–6). The CD19 antigen promptly took the spotlight as meeting the fundamental requirements for ideal CAR targeting, namely: its high expression on tumor cells, expression at all stages of B-cell development and thus on derived tumors, and absent expression on vital tissues, with the potential for B-cell aplasia considered a risk-benefit ratio worth taking (7). The other major advantage provided by hematological malignancies is their accessibility. CAR-T cells infused intravenously are much more likely to interact with tumor cells that are circulating in the blood or located in the bone marrow and lymph nodes (or lymphatic system), compared with the those that require penetrating through a solid tumor stroma.

Moving this technology to cancer treatment also required several advances in the laboratory. First, genetic modification of human cells became more mainstream, thanks to the development of efficient viral vectors, like lenti- and retro-virus based vectors, combined with the development of better protocols for vector production, cell transduction, and newer technologies for their safety monitoring (3, 8). Second, recognizing the need for a costimulatory signal (known as signal 2) to promote full T-cell activation was key. The provision of just the CD3ζ or CD3γ endodomains that trigger the proximal signaling of the TCR to the first-generation CAR was in fact unable to replicate the physiological sequence of activation events that occur upon TCR engagement and proved to be sub-optimal in supporting CAR-T cell expansion and persistence (9). In contrast, including the intracellular domains of molecules such as CD28 or 4-1BB in tandem with the CAR supported the transmission of signals capable of producing the required sustained activation, proliferation, and effector function (10–12).

## Informative first-in-human studies for advancing the field

**Figure 1:**
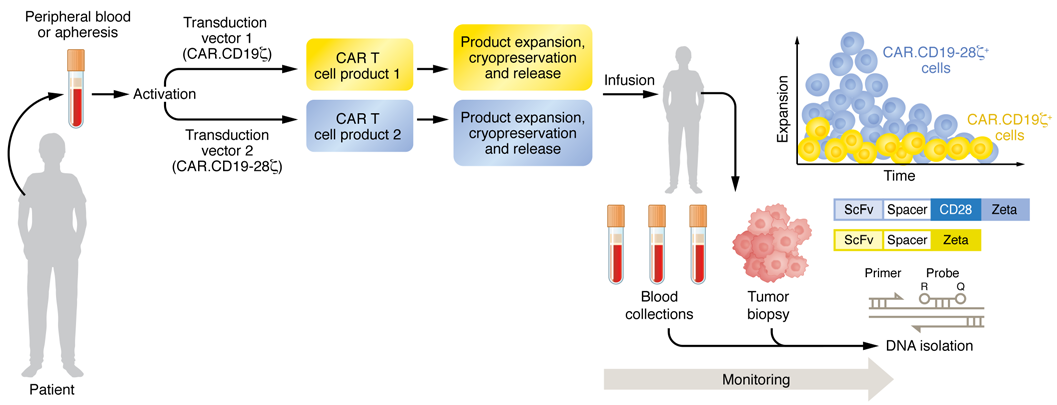
Simultaneous-infusion of distinct CAR-T cell products in the same patient reveals whether specific motifs support CAR-T cell expansion and function in patients with hematological malignancies. Peripheral blood was collected from patients with hematological malignancy, such as CD19^^+^^ NHL, and used as the source for T cells in the generation of two CAR-T cell products. Transduction of activated T cells was performed using two distinct vectors encoding the first generation and second-generation CAR.CD19, and CAR-T cells were expanded in parallel. The two CAR-T cells products were then co-infused in the patient, and blood and tumor samples were collected to detect each product. PCR can be used as an assay to monitor the kinetics of each product in patients. CAR-T cells encoding the CD28 signaling motif showed superior expansion and persistence in patients with CD19^^+^^ NHL (13).

To this end, in 2011 we conclusively demonstrated the essential role of co-stimulation in helping CAR-T cell expansion in vivo in the context of the human immune system (13). Our studies employed a simultaneous-infusion strategy, in which two CAR-T cell products were provided in the same patient. Each CAR-T cell product carried the identical scFv targeting CD19, but one included only the CD3ζ endodomain and the other included the CD28 costimulatory endodomain. These products were generated in parallel, starting from the same blood collection to further minimize any potential difference outside of CD28 co-stimulation. Although requiring the generation of two products for each individual, this study showed that the inclusion of the CD28 signaling motif supported superior activation, proliferation, and effector function of CAR-T cells in patients with CD19^+^ Non-Hodgkin lymphoma (NHL) (13). The major strength of the simultaneous-infusion study is that it removed from the evaluation all confounding factors, such as the heterogeneity of B-cell lymphomas, prior treatments, patient’s comorbidities or other intrinsic aspects, and allowed us to reach meaningful conclusions even with the small sample size that characterizes phase-I studies by allowing for comparisons within an individual patient (Figure 1).

We suggest that the design of small phase I clinical trials is also well poised to elucidate specific biological questions that remain critical in hematological malignancies. For example, we demonstrated that grafting the CD19 antigen on antigen-specific CAR T cells, such as virus specific T cells, yields alternative costimulatory signals when the native TCR of antigen-specific T cells engages with the cognate antigen expressed by effective antigen presenting cells (14). These types of biological implications, in fact, cannot be easily modelled in immune-deficient mice, which are typically obtained by engrafting with human tumors and treated with human CAR-T cells. Immune-deficient mouse models are also limited when used to address the even more complex setting of human diseases, such as the effects of the tumor microenvironment on the functionality of CAR-T cells. In light of these restrictions, immune-competent mouse models are being used more frequently, but differences in the evolutionary pathways and molecules, and requirements for tumor implant, rather than spontaneous development, may remain insufficient in recapitulating the clinical scenario. From our perspective, well-designed studies in humans also facilitate the reverse engineering process, or help with translation, informing us of where to go next. This bench-to-bedside, back-to-the-bench approach has proven instrumental to correlate prolonged survival of CAR-T cells enriched in naïve-central cells and/or memory-like cells, and helped us and others in implementing simple changes in manufacturing, like using different cytokines or enrichment protocols and shorter cultures (15, 16). One of these studies also underscored how responses can be better predicted if the infused product is further formulated with a defined ratio of CD4^+^ and CD8^+^ cells (16), a particularly important observation since the peripheral blood of patients with hematologic malignancies is usually characterized by variable proportions of these subsets due to prior therapies or underlying diseases.

Early phase CAR-T cell clinical trials have also addressed the importance of the conditioning therapy before CAR-T cell administration, even in patients with hematologic malignancies who are often fairly cytopenic. These studies demonstrated not only that conditioning therapy is needed (17, 18), but also that the inclusion of fludarabine in the lymphodepleting regimen optimize CAR-T cell expansion and persistence, likely because the addition of fludarabine promotes superior bioavailability of homeostatic cytokines, such like IL7 and IL15, to CAR-T cells and halts the cell-mediated elimination of CARs of murine origin (16, 18). As CAR-T cell therapies are being moved earlier in the treatment schedule, we consider these correlative analyses paramount. For example, patients will likely have received less chemotherapy, which may positively impact the quality of the infusion-product. On the other hand, when infused in the context of a more competent immune environment we will learn whether current lymphodepletion regimens will continue to prevent CAR immune-mediated rejection. Technologies advance at the speed of light and we should apply them not just to improve CAR design, but also to study in depth what happens to these cells upon infusion in patients, so we can create fitted cells for improved outcomes.

## Conclusions and future prospective

CAR-T cells have been transformative for B-cell acute lymphoblastic leukemias (ALL) and NHL. As we extend the benefits of CAR-T cells to other hematologic malignancies, new CARs and combinations are taking the stage, and additional challenges are emerging. More toxicities with new targets may occur when trying to differentiate between normal- versus tumor-specific antigens, since, as opposed to B-cell targeting, we cannot consider as acceptable risks the long-term myeloid aplasia or T-cell aplasia that could occur, for example, with CD33- or CD123-CARs for the treatment of acute myeloblastic leukemia, and with CD5- or CD7-CARs for T-ALL and NHL. Another evolving issue is tumor escape caused by antigen loss. Epitope loss has been reported with CD19 CAR-T cells, particularly in patients with leukemia (19), opening the need for multiple CAR-T cell targeting. However, lack of response durability after CAR-T cell treatment is most frequently caused by suboptimal CAR-T cells expansion and persistence, and by the presence of local inhibitory factors and/or cells (17, 18). Understating the exact cause calls for different combinatorial approaches. We believe that in-patient comparisons can be particularly handy when testing new strategies to overcome T-cell dysfunction, like persistence using dual costimulations (20) or co-expressing CARs and chemokine receptors to correct suboptimal trafficking of CAR-T cells to the tumor, as we are currently exploring in patients with Hodgkin lymphoma (NCT03602157).

As we reflect on the evolution of CAR-T cells over the past 30 years, with 6 CAR-T cell products approved by the FDA between 2017 and 2022, there is substantial anticipation that what we learned from CD19 CAR-T cells provides the foundations for expanding this approach to other hematologic malignancies and eventually to solid tumors. Setbacks or slow advances may pave the way in the solid tumor arena, but even if CAR-T cells do not become the holy-grail therapy, we still should not discount that this strategy has revolutionized how we treat B-cell malignancies and positively impacted the outcome of patients with hematologic malignancies.

## Version 1 01/16/2024

Electronic publication
